# Impact of ultrasound on extractability of native collagen from tuna by-product and its ultrastructure and physicochemical attributes

**DOI:** 10.1016/j.ultsonch.2022.106129

**Published:** 2022-08-20

**Authors:** Samaneh Pezeshk, Masoud Rezaei, Mehdi Abdollahi

**Affiliations:** a Department of Seafood Processing, Faculty of Marine Sciences, Tarbiat Modares University, P.O. Box 46414-356, Noor, Iran; b Department of Biology and Biological Engineering–Food and Nutrition Science, Chalmers University of Technology, SE 412 96 Gothenburg, Sweden

**Keywords:** Marine Collagen, Fish By-product, Ultrasound, Thermal stability, Tuna Skin

## Abstract

The effect of ultrasound treatment (300 W; 0, 5, 10, 15, 20 and 25 min) on the extractability of acid soluble collagen from yellowfin tuna skin and its structural, physicochemical and functional properties were investigated. Ultrasound treatments significantly increased collagen extraction yield from the tuna skin up to 2.7 times, compared to the conventional extraction with acetic acid. The level of proline, hydroxyproline and thermal stability of collagens increased by applying ultrasound while their native triple-stranded helical structure was well-preserved, as revealed by X-ray diffraction and FTIR spectroscopy. However, ultrasound treatment reduced the particle size of the collagens which increased their pH and salt induced solubility. The water holding capacity and the emulsifying properties of ultrasound treated collagens were also higher than those produced with the conventional method. Altogether, the results suggested that ultrasonication can be a promising assistant technology for improving native collagen extraction efficiency from tuna skin and its functionality but its duration should be carefully optimized.

## Highlights

• Collagen yield substantially increased with the aid of ultrasound.
• Ultrasonication did not disrupt the collagen native internal structures.
• Ultrasound increased denaturation temperature of collagen and its thermal stability.
• Functional properties of collagen recovered by ultrasound was superior to the control.

## Graphical abstract

**Figure:**
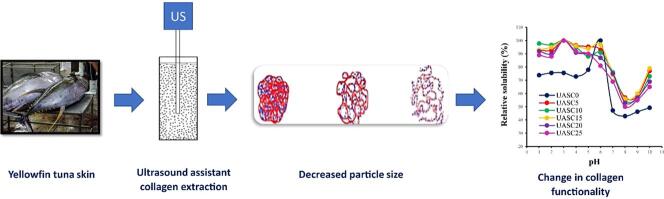

## 1 Introduction

The most abundant protein in vertebrates is collagen and it constitutes around 30 % of total protein content [1]. At least 28 kinds of collagens with dissimilar molecular, sequences and structures attributes have been identified, with collagen type I existence is the most usual form [2]. Collagen has very broad applications in food, pharmaceuticals, cosmetics, tissue engineering and biomedical products [3], [4]. Up to know, the main sources of collagen for the aforementioned applications are porcine and bovine bones and skins [2], [5]. Nevertheless, the prevalence of contagious and infectious diseases has raised concern among consumers about the use of animal-derived collagen [5]. Moreover, collagen derived from pigs is not acceptable owing to religious restrictions by Jews and Muslims [6]. Hence, alternative collagen resources such as fisheries by-products including scale, bone, skin and fin have gained great interest for collagen isolation. Marine resources present favorable properties of low immunogenicity, non-toxicity and also less religious limitations as a collagen source [7].

Fish skin generally contains a high percentage of collagen which account for about 30 % of it dry weight and is the most collagen rich part of fish [8]. The main methods conventionally used for extracting collagen includes acidic dissolution with or without enzymatic digestion which have been widely studied for several resources [9], [10]. However, the conventional extraction techniques normally result in a low collagen yield [11], [12] which calls for modifications and application of assistant technologies to increase collagen extraction efficiency.

Among the non-thermal assistant technologies ultrasonication has shown great potential for improving protein extraction efficiency and their functionality [13], [14], [15]. Generally, cavitation effects such as mechanical energy release, high pressure and local heat could improve protein extractability by accelerating phase transition or particle size reduction. It can also change molecular structure of protein by disrupting the intermolecular bonds such as electrostatic force and hydrogen bond which results in the modification of functional characteristics of protein [16]. Some studies have recently shown that the cavitation induced by ultrasound waves in liquid mediums have a significant effect on rate of collagen extraction as well as its physicochemical, structural and functional characteristics from bullfrog [17], soft-shelled turtle [14], golden carp [18], chicken cartilage [19] and chicken leg [20]. However, the effect of ultrasound on extraction efficiency of proteins and its optimum condition is very much dependent on the studied source and requires very careful evaluations. To the best of our knowledge there are very limited knowledge on the effect of ultrasound on the efficiency of collagen extraction from marine resource and especially tuna skin and its structural and functional properties.

Yellowfin tuna (*Thunnus albacares*) is one of the most important commercial fish species which is widely distributed in the tropical and subtropical areas of the Indian Ocean [21]. Yellowfin tuna is extensively utilized for production of canned tuna which generates a large amount of by-products which are either discarded as valueless materials or processed into fertilizer and low-value feed [22]. Bearing these in mind, the present study was aimed to evaluate the impacts of ultrasonication on the yield, ultrastructure and physicochemical attributes of collagen isolated from yellowfin tuna skin in combination with acidic extraction condition.

## 2 Materials and methods

### 2.1 Materials

Skin of yellowfin tuna (*Thunnus albacares*) was prepared by a seafood processing company in Sari, Iran and immediately (1 h) transferred to the laboratory. Non-fish skin components were removed by using a cutter on crushed ice, and the fish skin were washed with pre-cooling distilled water. Next, cut into little pieces (0.2 cm) and were packed into plastic bags. All raw materials were frozen and stored at −20 °C until their use. All reagents and chemicals used were of analytical grade.

### 2.2 Preparation of fish skin

The frozen tuna skins were first thawed at 4 °C, immersed into 0.1 M NaOH solution ratio of 1:10 (w/v) for 24 h to remove non-collagenous proteins. The NaOH solution was renewed at 8 h intervals, subsequently, the skins were washed with chilled distilled water until the wash water reached pH 7.0; then, subjected to degrease using butyl alcohol (10 % v/v) for 24 h with continuous stirring. The butyl alcohol solution was replaced every 12 h. Thereafter, defatted samples were washed with chilled distilled water three times to remove excess butyl alcohol.

### 2.3 Collagen isolation methods

#### 2.3.1 Non-ultrasound treated acid-soluble collagen

The isolation of acid soluble collagen **(**ASC) was named as non-ultrasonic treated acid-soluble collagen (UASC-0). The ASC was recovered from tuna skin based on the method described by Akram and Zhang (2020) [19] with slight modification. The pretreated skin was soaked into acetic acid (0.5 M) 1:30 w/v with continuous stirring at 4 °C for 48 h followed by centrifugation at 9000 × g for 45 min at 4 °C. The supernatant was collected and the remainder was re-recovered under the same manner. The supernatants were subsequently blended and salted out by the addition of NaCl to obtain a final concentration of 2.5 M in 0.05 M tris (hydroxymethylaminomethane), pH 7.0. The collagen precipitate, separated by centrifugation at 9000 g for 45 min, was solubilized in the minim volume of 0.5 M acetic acid, dialyzed (MWCO = 14 kDa) against acetic acid solution (0.1 M) for 12 h followed by distilled water until neutral pH was reached. The solution was replaced every 4 h and finally the collagen containing solution was lyophilized.

#### 2.3.2 Ultrasound treated acid soluble collagen

The pretreated skin was immersed to acetic acid 0.05 M (1:30, skin: acetic acid, w/v) and ultra-sonicated for 5, 10, 15, 20 and 25 min (off-time 3 s and on-time 2 s) using an ultrasonicator with a probe (Shanghai, DeYang, China) at a single frequency of 20 kHz and the power of 300 W, which named as UCSC5, UCSC10, UCSC15, UCSC20 and UCSC25, respectively. The solutions were placed under the ultrasound source (probe; diameter 1 cm) with a depth of 2.0 cm. the temperature was maintained at 3–5 °C using an ice-water bath [23]. Following ultrasonication, the samples were placed on a magnetized stirrer at 4 °C for 48 h for extraction. The subsequent steps were done as explained above (section 2.3.1).

### 2.4 Collagen yield

The yield of collagen was calculated using equation number 1:

(1) $$Yield\%=\frac{m1}{m2}*100$$

where m1 was the dry tuna collagen weight and m2 was the wet tuna skin weight.

### 2.5 Amino acid composition

Amino acid composition was evaluated as explained by Song et al. (2021) [7]. Briefly, the collagen samples were blended and hydrolyzed for 22 h in 6 M HCl at 110 °C. The hydrolysates were evaporated, the residual contents were solved in citric acid and analyzed using an amino acid analyzer (L8900, HITACHI, Japan).

### 2.6 UV absorption

The collagen UV absorption spectrum was evaluated based on the method explained by Abdollahi et al. [24]. Freeze-dried collagen samples were dissolved in 0.5 M acetic acid (0.5 g/l) and its UV–visible spectrum was recorded using a spectrophotometer (UV-1800, Mapada Instruments Co., Ltd., Shanghai, China) from 200 to 320 nm.

### 2.7 SDS-Page

SDS-PAGE was conducted as previous method illustrated by Zhu et al. [25]. The concentrations of the separating and stacking gels were 12 % and 5 %, respectively. The collagens were dissolved in sample buffer (2 % SDS, 0.0625 mol L ^-1^ Tris HCl, 0.01 % bromophenol blue, 10 % glycerol, and 5 % 2-mercaptoethanol) at a ratio of 1:2 (w/v). Then, the samples were heated for 5 min at 95 °C and 10 μL of protein were applied to each lane. The running voltage varied from 100 V to 200 V. After electrophoresis, the gels were stained with Coomassie Brilliant Blue R-250 and destained with methanol (50 %) and acetic acid (10 %) until clear bands were achieved.

### 2.8 Fourier transform infrared spectroscopy

FTIR spectra of collagens were obtained using an infrared spectrophotometer (Bruker, Karlsruhe, Germany) at 25 °C with resolution 2 cm^−1^ (range 4000–500 cm-1). Each sample was prepared by mixing the freeze-dried collagen (10 mg) with KBr and then pressing it into a translucent pellet using a hydraulic press. The mode of vibrations and functional group were recognized based on the peak of interest at a specific wavelength and absorbance [4].

### 2.9 X-ray diffraction analysis

The X-ray diffraction analysis of the collagen samples were obtained by X-ray diffraction instrument (Bruker, Germany). Its radiation wavelength was λCukα1 = 1.5406 Å. The 2θ range was from 5° to 35°, the X-ray source was Cu Ka, and the scanning speed was 2°/min [2].

### 2.10 Thermal stability

Thermal stability of collagens was characterized by a differential scanning calorimeter (DSC) (Perkin Elmer, Norwalk, CA, USA). The collagen powder was rehydrated with deionized water at a solid to solution ratio of 1:40 (w/v) and equilibrated at 4 °C for 24 h and heated from 20 to 100 °C at a rate of 10 °C min^−1^, according to the method described by Kaewdang et al. [26].

### 2.11 Viscosity

The viscosity of collagen solution (0.03 % w/v) in 0.1 M acetic acid was determined using a viscometer (Brookfield Engineering Laboratories ltd., Middleboro, USA) and a spindle n. 1 at a speed of 100 rpm. Solutions were heated with a heating rate of 20 to 45 °C. The temperature of collagen solutions (10 mL) ramped up stepwise and maintained for 30 min at each chosen temperature. The relative viscosity was determined relative to viscosity obtained at 20 °C. The denaturation temperature, T_d_, was measured as the temperature that the change in viscosity was half completed [1].

### 2.12 Particle size

The particle size of collagen was evaluated using a particle size analyzer (Malvern, UK). The collagen samples (1 mg/ml) were dispersed in acetic acid (0.5 M) and subjected to particle size measurement. All measurements were repeated three times [14].

### 2.13 Scanning electron microscopy

The morphological properties of lyophilized collagen samples were observed using a scanning electron microscope (SEM) (*SEC*, Mini-SEM SNE-3200 M, South Korea). The collagen samples were mounted onto stub of copper with a double-sided adhesive tape and observed at magnification of 2000 × and at an accelerating voltage of 15 kV.

### 2.14 Effect of pH on collagen solubility

The solubility of collagen solutions (3 mg/mL collagen) was evaluated following the method described by Shaik et al. (2021) [27]. The pH of collagen solution was adjusted with 6 mol/L HCl or 6 mol/L NaOH to 1 to 10. The volume of protein solution was brought to 10 mL with cold distilled water at the corresponding pH. The solutions were stirred at 4 °C for 20 min and centrifuged at 9,000 rpm for 20 min at 4 °C. The protein content in the supernatant was determined using Lowry method as referred by Markwell et al. (1978) [28].

### 2.15 Effect of NaCl on collagen solubility

The impact of NaCl concentrations of 0 %, 1 %, 2 %, 3 %, 4 %, 5 %, and 6 % on the solubility of isolated collagen was conducted following the method described by Tan et al. [8]. Collagen solutions (6 mg/mL) were centrifuged at 9,000 rpm for 20 min at 4 °C. The protein concentration in the supernatant was measured as previously described.

### 2.16 Water holding capacity

The water-holding capacity (WHC) was characterized as described by Li et al. [12]. Briefly, 0.5 g of the collagen sample and 2 mL of distilled water were added into a weighed culture dish (d = 10 cm). It was then put into an incubator with a temperature of 37 °C and a relative humidity of 75 %. The dish was weighed every 10 min in 70 min. WHC was calculated with the following formula (2):

(2) $$WHC\left(\%\right)=\left(\Delta W/W0\right)\times 100$$

where ΔW is the change in the amount of water at each time and W0 is the initial amount of water in the culture dish.

### 2.17 Emulsifying properties

To begin with, 9 mL of 2 g/L collagen solutions were mixed with 3 mL corn oil and homogenized using Ultraturax (T25D, IKA, Germany) at 10,000 rpm for 1 min. Then, 50 μL of the emulsion was pipetted from the vessel and diluted in a beaker with 5 mL of 0.1 % sodium dodecyl sulfate solution. The emulsifying activity index (EAI) and emulsion stability index (ESI) of the collagens were determined as explained by Pezeshk et al. [23].

### 2.18 Statistical analysis

The results were analyzed using one-way analysis of variance in SPSS Statistic (Version 20.0 for Windows). The data were expressed as the mean ± standard deviation of triplicates. Comparison of treatment means was based on Duncan’s multiple range test. Differences with a probability value of p < 0.05 were defined as significant.

## 3 Results and discussion

### 3.1 Yield of collagen

Yield of collagen produced using the different treatments are summarized in Table 1. Acid-soluble collagen was isolated from tuna skin using the conventional method with yields of 5.47 % and 21.49 % (wet and dry weight basis, respectively). The skin’s collagen was not totally solubilized with 0.5 M acetic acid extraction. This finding was in accordance with recently reports on channel catfish skin [8] and golden carp skin [18] who stated the incomplete solubilization of collagens in fish skin in 0.5 M acetic acid. Low yield of collagen extraction using the acid could suggest that there were many cross-links by covalent bonds at the telopeptide region of the collagen that resulted in a low solubility of collagen [24]. By application of ultrasound, collagen extraction yield was remarkably (p < 0.05) enhanced as ultrasound time increased compared to the control. Hereby, the highest yield of collagen was achieved with ultrasound treatment for 25 min (14.53 %; wet weight basis and 57.06 % dry weight basis). The observed improvement in yield with application of longer ultrasonication time may be related to cavitation phenomena inducing cell disruption and the rendered mechanical energy which increases penetration of acid into the matrix of the skin for extraction of collagen [27]. These findings are in agreement with those previously reported when ultrasonication applied on clown featherback skin [29] and chicken sternal cartilage [19] which remarkably increased collagen extraction yield compared with the conventional method.

**Table 1:** Yield of acid soluble collagen-I from the yellowfin tuna skin through different time of ultrasound (0 min: UASC0, 5 min: UASC5, 10 min: UASC10, 15 min: UASC15, 20 min: UASC20 and 25 min: UASC25).

Tissue	Moisture (%)	Collagen	Yield (%)	
			Wet weight basis	Dry weight basis
Skin	75.51 ± 0.5	UASC-0	5.47 ± 0.43^f^	21.49 ± 48^f^
		UASC-5	6.33 ± 0.5^e^	24.86 ± 0.7^e^
		UASC-10	9.42 ± 0.1^d^	37.01 ± 0.11^d^
		UASC-15	9.7 ± 0.14^c^	37.95 ± 0.12^c^
		UASC-20	10.02 ± 0.83^b^	39.37 ± 0.95^b^
		UASC-25	14.53 ± 1.1^a^	57.06 ± 0.9^a^

Values are presented as mean ± SD.

Different letters in the same column are significant differences at p < 0.05.

### 3.2 Amino acid composition

Table 2 illustrates amino acid composition of the collagens extracted from tuna skin. Glycine was the most abundant amino acid in all tuna skin collagens, which is necessary for the formation of a helical structure [30]. Furthermore, the glutamine, alanine, hydroxyproline and proline contents were high in all samples, whiles the tyrosine, threonine, isoleucine, methionine and histidine contents were relatively low. These findings are in agreement with the amino acid composition reported for collagen extracted from nile tilapia [7], grass carp [24], bigeye tuna [31], and walleye pollock [1].

**Table 2:** Amino acid contents of acid soluble collagen-I from the yellowfin tuna skin by different time of ultrasound (0 min: UASC0, 5 min: UASC5, 10 min: UASC10, 15 min: UASC15, 20 min: UASC20 and 25 min: UASC25) (residues/1000 amino acid residues).

Amino acids	UASC-0	UASC-5	UASC-10	UASC-15	UASC-20	UASC-25
Alanine	118.2 ±.02^a^	110.3 ± 0.2^c^	99.5 ± 00.3^e^	95.1 ± 0.3^f^	108.2 ± 0.21^d^	115.3 ± 0.22^b^
Arginine	60.3 ± 0.3^b^	54.3 ± 0.2^d^	51.2 ± 0.4^f^	52.6 ± 0.4^e^	59.7 ± 0.3^c^	64.8 ± 0.4^a^
Asaragine	45.2 ± 0.3^a^	41.3 ± 0.5^c^	34.4 ± 0.4^d^	30.5 ± 0.3^e^	44.4 ± 0.3^b^	44.4 ± 0.2^b^
Cysteine	0	0	0	0	0	0
Glutamine	111.5 ± 0.22^c^	110.7 ± 0.3^d^	99.4 ± 0.2^e^	98.5 ± 0.1^f^	113.6 ± 0.3^b^	126.6 ± 0.4^a^
Glycine	269.5 ± 0.7^a^	260.7 ± 0.6^c^	245.7 ± 0.9^e^	234.9 ± 0.4^f^	252.6 ± 0.8^d^	264.5 ± 0.4^b^
Histidine	13.2 ± 0.1^b^	11.1 ± 0.1^d^	12.2 ± 0.3^c^	10.3 ± 0.05^e^	12.2 ± 0.5^c^	15.4 ± 0.3^a^
Isoleucine	12.4 ± 0.3^b^	11.2 ± 0.2^c^	11.1 ± 0.1^c^	11.09 ± 0.22^c^	11.09 ± 0.12^c^	13.2 ± 0.32^a^
Leucine	31.5 ± 0.5^b^	28.4 ± 0.45^d^	27.5 ± 0.54^e^	24.5 ± 0.44^f^	29.4 ± 0.3^c^	32.3 ± 0.3^a^
Lysine	35.5 ± 0.2^a^	33.3 ± 0.4^b^	26.3 ± 0.2^e^	23.6 ± 0.11^f^	31.4 ± 0.33^c^	30.5 ± 0.5^d^
Methionine	9.07 ± 0.1^d^	9.09 ± 0.1^d^	10.1 ± 0.3^c^	7.09 ± 0.11^e^	12.1 ± 0.12^b^	14.1 ± 0.1^a^
Phenylalanine	26.3 ± 0.3^a^	25.5 ± 0.4^c^	20.3 ± 0.44^d^	17.2 ± 0.33^e^	25.6 ± 0.23^c^	25.4 ± 0.12^b^
Hydroxyproline	96.9 ± 0.5^d^	99.6 ± 0.45^b^	90.8 ± 0.43^e^	82.7 ± 0.5^f^	97.8 ± 0.5^c^	101.7 ± 0.6^a^
Proline	90.5 ± 0.34^d^	93.5 ± 0.4^b^	85.6 ± 0.2^f^	85.7 ± 0.3^e^	91.4 ± 0.4^c^	96.7 ± 0.4^a^
Serine	37.4 ± 0.5^a^	37.6 ± 0.56^a^	30.3 ± 0.3^d^	29.2 ± 0.1^e^	34.1 ± 0.2^c^	36.3 ± 0.3^b^
Threonine	11.2 ± 0.1^d^	19.5 ± 0.1^a^	17.2 ± 0.12^b^	17.1 ± 0.12^b^	15.5 ± 0.13^c^	15.5 ± 0.1^c^
Tyrosine	4.1 ± 0.04^b^	4 ± 0.05^b^	3 ± 0.1^c^	2 ± 0.11^d^	4 ± 0.04^b^	5 ± 0.12^a^
Valine	26.2 ± 0.12^a^	25.5 ± 0.3^b^	20.1 ± 0.5^c^	17.2 ± 0.4^d^	26.3 ± 0.4^a^	25.4 ± 0.3^b^
Imino acid*	187.4 ± 0.42^d^	193.1 ± 0.42^b^	176.4 ± 0.32^e^	168.4 ± 0.4^f^	189.2 ± 0.45^c^	198.4 ± 0.5^a^

Values are presented as mean ± SD.

Different small letters in the same raw are significantly (p < 0.05) different.

Hydroxyproline and proline are unique amino acids in collagen [5]. Overall, the proline and hydroxyproline contents of sonicated collagens increased compared with UASC-0 as the ultrasonication time increased. However, ultrasonication for 10 and 15 min resulted in decrease in the content of proline and hydroxyproline in the collagens. The content of proline and hydroxyproline in the tuna skin collagens extracted here was similar to those reported in nile tilapia (165–186/1000 residues) [7], grass carp (161–181/1000 residues) [10] and ocellate puffer (170/1000 residues) [30] but lower than that of other vertebrates' collagen [5]. This may be attributed to the difference in their species and habitats [5], [10]. In addition, a higher content of proline and hydroxyproline led to a higher thermal denaturation temperature, better thermal stability and stronger mechanical resistance of collagens. This strengthens the helix structure because of the formation of pyrrole rings and hydrogen bonds [32].

### 3.3 UV absorbance

**Fig. 1:**
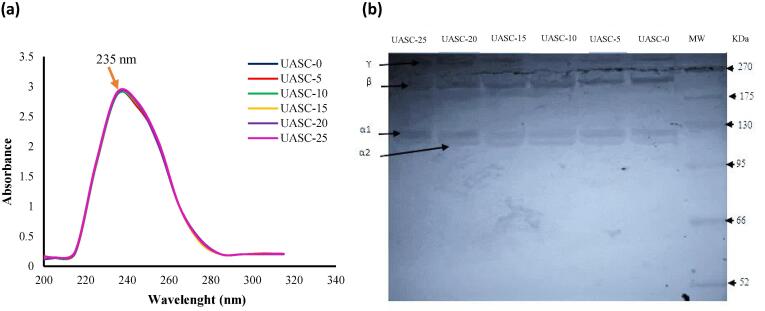
UV absorption spectra (a) and SDS-PAGE profiles (b) of acid soluble collagen collagen extracted with the aid of ultrasound at different treatment time (0 min: UASC0, 5 min: UASC5, 10 min: UASC10, 15 min: UASC15, 20 min: UASC20 and 25 min: UASC25) from yellowfin tuna skin.

The ultraviolet absorption spectra of collagen samples are shown in Fig. 1a. Maximum absorption peaks of all treatments were observed at around 235 nm, primarily attributed to the presence of –COOH, CONH_2_ and C=O groups in the polypeptides chains of the produced collagens [33]. This absorption peak is commonly acquired between 220 and 280 nm [24]. Also, it is noteworthy that any other maximum peaks were not displayed at 280 nm. This is attributed to the low content of tyrosine in collagen samples which absorb UV light at 280 nm [34]. Hence, all the collagens were isolated with minimum amount of contamination with other types of protein.

### 3.4 Polypeptide patterns

Fig. 1b shows polypeptide patterns of all collagens from yellowfin tuna skin. All collagens were comprised of α1- and α2 chains of around 122 and 118 kDa, respectively, and their dimer (β-chains), and small amounts of trimer (γ-chains). The findings showed that ultrasonication treatment did not negatively affect on polypeptide patterns of tuna skin collagen, in terms of intensity or Mw of each band. In general, the ratio of α1 to α2 chains were about 2:1 in all recovered collagens extracted from yellowfin tuna skin that result is compatible with type I collagens classification [35]. It is not possible to conclude whether all of the samples examined comprise the α3 chain based on the obtained electrophoretic patterns since the α1 chain cannot be separated from α3 chain under the electrophoretic conditions due to their similar peptide migration and chemical nature [5].These electrophoretic patterns of tuna skin collagen were similar to those of golden carp skin collagen [18], walleye pollock skin collagen [1] and collagen of bigeye snapper [36]. The distribution of collagen molecular weight will affect its quality, while the observation of β and α component showing that the all collagen treatments were pure and their structure integrity were maintained during ultrasonication application [12] and no significant differences were detected in protein bands amongst the all isolated collagens.

### 3.5 FTIR

**Fig. 2:**
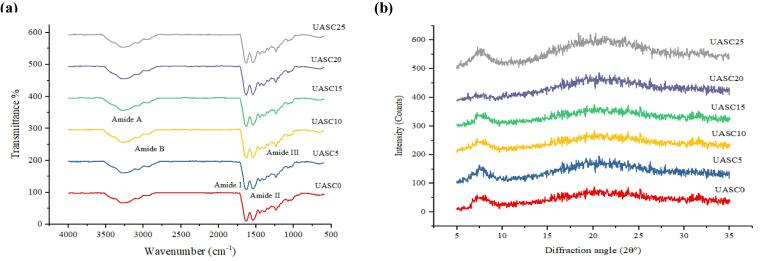
FTIR spectra (a) and XRD patterns (b) of acid soluble collagen extracted by different time of ultrasound treatment (0 min: UASC0, 5 min: UASC5, 10 min: UASC10, 15 min: UASC15, 20 min: UASC20 and 25 min: UASC25) from yellowfin tuna skin.

Fig. 2a shows the FTIR spectra of the collagen samples. The amide A peaks of USAC-0, USAC-5 and USAC-10-25 (USAC-10, USAC-15, USAC-20 USAC-25) were found at 3332, 3311 and 3301 cm^−1^, respectively. Amide A usually occurs between the ranges of 3400–3440 cm^−1^, however it might slightly move to lower wavenumbers while the NH group of a protein forms intermolecular hydrogen bond [17]. It seems that the presence of more hydrogen bonds interactions amongst the some of the treated collagen molecules (USAC-10, USAC-15, USAC-20 and USAC-25) compared to the other collagen samples, thence, resulting to transferring to lower wavenumber. The amide B peak position of collagen samples was found at 2933 cm^−1^, which is consistent with unsymmetrical stretch of CH_2_ [37]. The amide I peaks of samples were measured at 1627 cm^−1^, which is attributed to stretching vibrations of C=O coupled with N—H bond. Generally, it is associated with protein secondary conformational and is often found in the range of 1600–1700 cm^−1^ [38]. Amide II bands of samples were appeared at 1544.77 cm^−1^, which may be attributed to N—H bending and C—N stretching [39]. The amide III absorption peak often observes between 1220 and 1320 cm^−1^ and reflects the N—H deformation and the C—N stretching, which is responsible for triple helical structure of collagen [40]. The triple- helix conformational of the collagen is kept usually by limitations on changes in the secondary conformational of the polypeptide string by the pyrrolidine rings [12]. In this study, all the isolated collagens exhibited amide III absorption peaks at 1234 cm^−1^. In addition, the ratio of absorption between the amide 3 band and CH2 bend (1430 cm^−1^), about 1.0, approved that triple helical conformational of the collagen samples were still stable in UASC0 and ultrasound-treated acid soluble collagens.

### 3.6 X-ray diffraction

The XRD patterns of collagen samples are shown in Fig. 2b. All samples had two typical peaks related to collagen structure at around 2θ of 7.5°and 21.2° [41]. The d Å (minimum value) of the repeat spacings is determined using Bragg equation d (Å) = λ/2sinθ (λ = 1.54 Å) [42]. The d value of 7.57° (the first sharp peak) was 11.6 Å that is attributed to the triple helix conformation of collagen, showing the distance among molecular chains. The d values of 21.2° (the second broad peak) was 4.1 Å which is related to the distance among skeletons and is similar to acid-soluble collagen of nile tilapia skin (11.56 Å and 4.41 Å) [2] and red stingray skin (11.1 Å and 4.5 Å) [43]. Generally, the results suggested that all isolated collagens maintained a native internal structure and undenatured and application of ultrasound did not effect on collagen structural integrity.

### 3.7 Differential scanning calorimetry

**Fig. 3:**
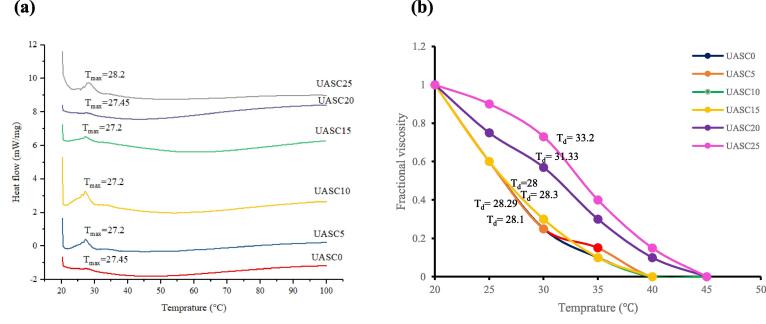
DSC profiles (a) and fractional viscosity (b) of acid soluble collagen extracted with the aid of ultrasound at different treatment time (0 min: UASC0, 5 min: UASC5, 10 min: UASC10, 15 min: UASC15, 20 min: UASC20 and 25 min: UASC25) from yellowfin tuna skin.

Fig. 3a shows the DSC profiles of collagen samples. T_max_ are referred to the temperature at which the collagen fiber was shrunk to one third of its length and the physical form of collagen was transformed from solid to liquid [19]. In this study, the T_max_ values of collagens isolated are ranging from 27.2 up to 28.2 °C for UASC0 and UASC25, respectively. The T_max_ of collagen extracted decreased gradually with the increase in the ultrasonication time from 0 to 15 min which might be attributed to cavitation intensity and turbulence produced by the ultrasound probe. These energies could disrupt the covalent crosslinks between the collagens. However, with the more increase in the ultrasonication time from 15 to 25 min, there was an enhancement in thermal stability of collagen samples. This means prolonging the ultrasound treatment might have enhanced the aggregation of the collagen particles by the creation of different types of covalent bonds [23]. Moreover, the thermal stability of collagen is straightly positive related to the contents of proline and hydroxyproline via their intermolecular cross-linking [19] which is in line with the higher content of proline and hydroxyproline found in UASC25 compared with the control (see Table 2). T_max_ of protein samples was lower than those of collagen from skin of calf at 40.07 °C [6] and was greater than that collagen sample from skin of Spanish mackerel at 15.12 °C [12]. These differences in thermal stability among collagens from difference species have been related to the different contents of proline and hydroxyproline, environmental temperature and body temperature [44].

### 3.8 Viscosity

Denaturation temperature (T_d_) are referred to the temperature at which the collagen triple helix conformational breaks down into random coils. The fractional viscosity of collagen samples under a temperature range of 20–44.5 °C shown in Fig. 3b. The fractional viscosity of the samples reduced as the temperature enhanced and T_d_s obtained were 28.1, 28.29, 28.3, 28, 31.33 and 33.2 °C, for UASC0, UASC5, UASC10, UASC15, UASC20 and UASC25, respectively. T_d_ value of UASC0 was lower than the T_d_ of pig skin collagen [30]. These findings more demonstrated that the triple helix structure of collagen from yellowfin tuna skin was less thermal stable than those of land animals collagen and this was more related to the animal body temperature and the temperature of their habitat. Compared to the denaturation temperatures of those obtained from other marine animals, T_d_ was similar to collagens from the puffer fish skin (28 °C) [30] and of paper nautilus skin (27 °C) [45]. Generally, collagen T_d_ results could be related to the content of Pro and Hyp [9], and in this regard, UASC25 sample with T_d_ equal to 33.2 °C contained a higher content of proline and hydroxyproline, compared with other treatments (197 residues/1000 residues). The T_d_ measurements for the different collagen samples were also in line with the T_max_ measurements obtained with DSC (see Fig. 3a), which showed that with increasing ultrasonication time from 15 to 25 min induced some changes in the collagen helical structures. The collagen structural stability is necessary for its potential uses in pharmaceutical and biomedical products.

### 3.9 Particle size

**Fig. 4:**
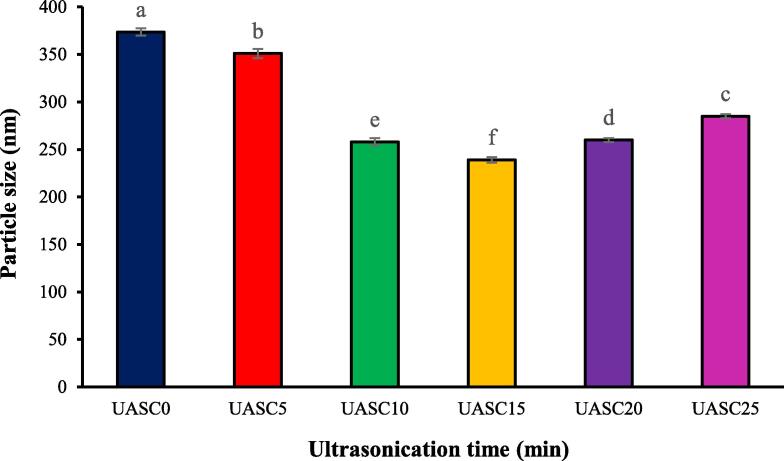
Particle size of acid soluble collagen extracted with the aid of ultrasound at different treatment time (0 min: UASC0, 5 min: UASC5, 10 min: UASC10, 15 min: UASC15, 20 min: UASC20 and 25 min: UASC25) from yellowfin tuna skin. Different letters indicate significant differences at *p* < 0.05.

The size of protein is one of the main factor, which could affect the functional and biopharmaceutical characteristics of the protein [46]. The impact of the ultrasound treatment on the collagens’ particle size is shown in Fig. 4. The particle size of collagen reduced with the enhancement in the ultrasound time from 0 to 10 min (p < 0.05), which was maybe caused by the cavitation of particles under ultrasonication [15]. The mechanical and cavitational forces would interrupt the non-covalent interaction among collagens, resulting the decreased collagens size [14]. However, with the more increase in the sonication time (20 and 25 min), there was a remarkable enhancement in collagen particle size, stating that prolonging the ultrasound enhances the particles aggregation by the creation of different types of covalent bonds [22]. Therefore, the control of surface characteristics of reduced particles is important and optimized ultrasonication condition should be determined.

### 3.10 SEM

**Fig. 5:**
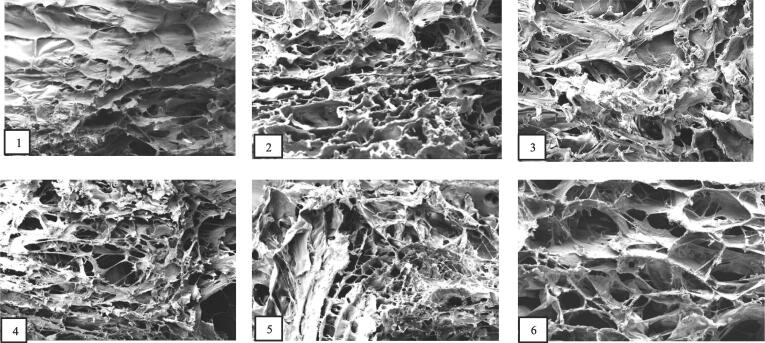
SEM images of acid soluble collagen extracted with the aid of ultrasound at different treatment time (0 min: 1, 5 min: 2, 10 min: 3, 15 min: 4, 20 min: 5 and 25 min: 6) from yellowfin tuna skin at 2000x magnification.

The microstructures of collagen samples was observed using SEM (Fig. 5). The illustration shows irregular dense sheet-like film microstructure which is typical for purified collagens. As can be seen, the UASC0 showed an uneven, dense and multilayered aggregated structure and a wrinkled surface possibly due to dehydration during drying [14]. Nevertheless, microstructure of the ultrasound treated collagens were more porous and homogenous with a meshwork appearance. These porous patterns of collagens were observed clearer as ultrasonication treatment time increased due to the mechanical fluctuations and cavitation of the ultrasound wave [27]. In UASC25, the prolonged ultrasound time affected the structure and length of the fibrils. The findings concluded that ultrasonication induced apparent physical changes in the microstructure of acid soluble collagen from tuna skins.

### 3.11 Solubility

**Fig. 6:**
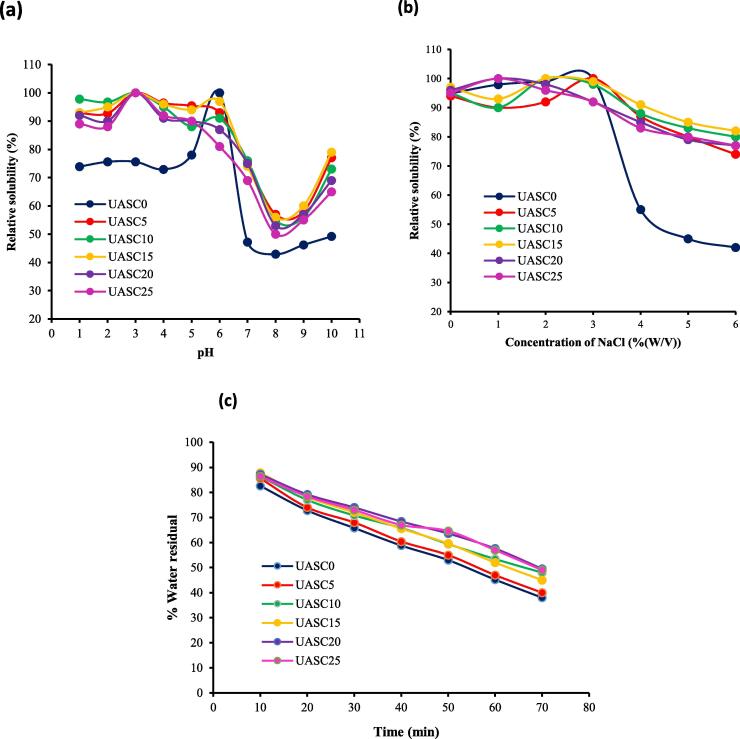
Relative solubility (%) at different pH (a) and NaCl concentrations (b) and water-holding capacity (c) of acid soluble collagen extracted with the aid of ultrasound at different treatment time (0 min: UASC0, 5 min: UASC5, 10 min: UASC10, 15 min: UASC15, 20 min: UASC20 and 25 min) from yellowfin tuna skin.

Solubility of all collagens produced from yellowfin tuna skin in the pHs of 1–10 is illustrated in Fig. 6a. All samples extracted showed high relative solubility in acidic conditions (pH < 6), with the highest solubility at pH 3 and 2 for ultrasound treatments and control sample, respectively. A considerable reduction in solubility of the collagen extracted was detected at pH 6–8. The significant decrease in solubility within the isoelectric point range of collagen (pH 6–9) might be due to hydrophobic-hydrophobic interactions between protein molecules, which causes collagen precipitation [11]. However, the solubility of collagens slightly increased at pH 8–10. This phenomenon could be related to the repulsive effect amongst polypeptide chains at pHs above isoelectric point [6]. Noticeably, the relative solubility of collagens extracted with ultrasound treatments was higher than control sample at all studies pH values which might be related to a lower degree of crosslinking, weaker bonding and smaller particle size in collagens extracted with the aid of ultrasound treatments compared with UASC0 [12]. Similarly, Zou et al. [14] stated that ultrasound treatment can increase relative solubility of acid-solubilized collagen from soft-shelled turtle.

Fig. 6b shows the solubility of all collagens extracted at the NaCl concentrations of 0–6 %. The solubility of samples stayed high up to NaCl of 3 % (w/v). The relative solubility of all collagens isolated considerably reduced at 4 % (w/v) NaCl and above. These findings are similar to earlier studies on collagen isolated from skin of striped catfish [36] and brown stripe red snapper [4] which stated that solubility of collagens reduced with the increasing NaCl concentration. At higher concentrations of NaCl (more than 3 %), hydrophobic sites are exposed by salt ions due to the loss of hydration shell on the collagen surface. This leads to increased hydrophobic interactions among the chains of the protein resulting in collagen precipitation [40]. Collagen isolated by ultrasound treatment showed higher solubility than the control sample (UASC0) at NaCl concentrations of 4–6 % (w/v). This might have been attributed to smaller particle size and the differences in structures and amino acid compositions between ultrasound treatments and UASC0.

### 3.12 Water-holding capacity

Water-holding capacity (WHC) of protein could be influenced by many factors including ionic strength and concentration of protein [27]. As can be seen in Fig. 6c, the WHC of ultrasound treatments enhanced with increasing ultrasonication time, compared with the convectional acid extraction. This could be related to the the physicochemical and microstructure characteristics of collagens such exposure of more hydrophilic and charged groups induced by application of ultrasound [47]. In agreement with this study, Zou et al. (2017) [14] reported that WHC of turtle calipash collagen improved by the ultrasound treatment. Akram and Zhang (2020) [19] also reported higher values WHC for chicken sternal cartilage collagen after being treated with ultrasound, compared with the collagen isolated by pepsin (control). Generally, collagens extracted with the aid of ultrasound treatment showed a good water absorption and maintenance ability which could be beneficial for their application in cosmetic, food and pharmaceutical industries.

### 3.13 Emulsion activity and emulsion stability

**Fig. 7:**
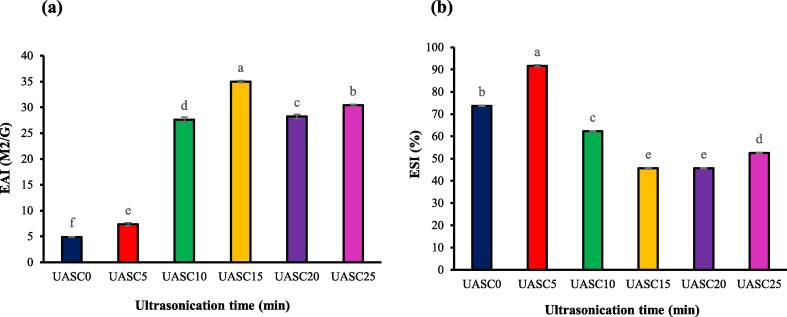
EAI (a) and ESI (b) of acid soluble collagen extracted with the aid of ultrasound at different treatment time (0 min: UASC0, 5 min: UASC5, 10 min: UASC10, 15 min: UASC15, 20 min: UASC20 and 25 min: UASC25) from yellowfin tuna skin. Different letters indicate significant differences at *p* < 0.05.

The emulsifying activity index (EAI) and emulsifying stability index (ESI) of acid soluble collagen were remarkably effected (p < 0.05) by ultrasonication treatment. The EAI demonstrates the ability of proteins to participate in the formation of emulsions [23]. The emulsification characteristics of collagen was changed by changing ultrasonication time. As can be seen, the EAI of collagens remarkably (p < 0.05) improved after using ultrasonication compared to the control (Fig. 7a). The highest EAI of the collagen achieved using ultrasound treatment for 15 min. These findings are also supported by observed trend in our results for the collagen particles size (Fig. 4), which showed the lowest particle size for the collagen isolated using ultrasound treatment for 15 min. Similar results about the modification of protein emulsion activity by sonication were found for chicken cartilage collagen [19] and fish proteins [22]. Those studies stated that the improvements in EAI of proteins exposed to sonication could be related to enhance in solubility and decrease in their particle size and their structural unfolding induced by ultrasonication treatment. As can be seen from Fig. 7b, the ESI of the collagens increased (p < 0.05) as the sonication time increased from 0 to 5 min (300 W) which was probably due to formation of smaller droplets in the emulsions produced with collagens sonicated for 5 min [26]. However, as the ultrasonication time increased from 5 to 25 min, the ESI of collagens isolated significantly decreased (p < 0.05), in comparison with the control treatment. This could be due to the changes in collagens lipophilic-hydrophilic arrangements or molecular length at too low pH [23].

## 4 Conclusion

The isolation of native collagen from yellowfin tuna skin was successfully performed with the aid of ultrasound. The collagen yield enhanced remarkably (p < 0.05) as the time of ultrasonication increased. The proline and hydroxyproline contents of the ultrasound treated collagen was higher than those recovered using the conventional method. Ultrasonication also improved the thermal stability, functional and physicochemical characteristics of collagen which might be related to some changes in the collagen helical structures and decreased collagens particle size but it did not affect on the structural integrity and molecular stability of collagens. Furthermore, all collagen samples produced from yellowfin tuna skin had high solubility at NaCl concentrations <3 % (w/v) and pH 1–4. These results suggest that the ultrasound treatment could be a beneficial method to assist collagen extraction from fish skin resulting in both higher collagen extraction yield and functionality with better process efficiency and practical application potentials.

## Declaration of Competing Interest

The authors declare that they have no known competing financial interests or personal relationships that could have appeared to influence the work reported in this paper.

## Data Availability

Data will be made available on request.
